# Assessment of the Biosafety and Biosecurity in the Reference Veterinary Laboratory of Parakou in Benin

**DOI:** 10.3390/tropicalmed6030146

**Published:** 2021-08-04

**Authors:** Vincent Dossou Sodjinou, Paul Ahoumènou Ayelo, Agué Germain Aïndé Achade, Dissou Affolabi, Dona Edgard-Marius Ouendo

**Affiliations:** 1 Regional Institute of Public Health (IRSP), University of Abomey-Calavi, 01BP 918 Ouidah, Benin; eouendo@irsp-ouidah.org; 2 Medical Sciences Faculty, University of Abomey-Calavi, 01BP 188 Cotonou, Benin; paulayelo@yahoo.fr (P.A.A.); affolabi_dissou@yahoo.fr (D.A.); 3 Livestock Direction, Ministry of Agriculture Livestock and Fisheries, 01BP 2041 Cotonou, Benin; germachade@yahoo.fr

**Keywords:** biosafety, biosecurity, global health security

## Abstract

Optimal biosafety and biosecurity are major requirements of global health security. This study assessed the biorisk management in the reference veterinary laboratory of Parakou (Benin). The study was cross-sectional, descriptive, and evaluative. The non-probability sampling method with the reasoned choice was used. The Food and Agriculture Organization laboratory mapping tool-safety was used to collect information from the laboratory team. Group discussion, working environment observation, and document exploitation were the data collection techniques. The biorisk management was rated good if the average indicator of the laboratory reached at least 80%. Otherwise, the biorisk management was rated insufficient. The overall laboratory biosafety and biosecurity score was insufficient (42.4%). Per area, the scores were 26.7% for engineering, 33.3% for administration, 53.8% for personal protective equipment, and 62.3% for the operational. There was no area or category score that reached 80%. Containment, waste disposal, and personal protective equipment disposal were the best performing categories with a score above 60%. The laboratory has no biosafety and accident prevention program. Its premises require renovation. The standard operating procedures for biosafety are not yet finalized, and the training mechanism is not optimal. Therefore, strong advocacy and implementation of a biorisk management improvement plan appear as urgent corrective actions which are required to help the reference veterinary laboratory of Parakou in its task to protect the livestock and, ultimately, the people of Benin from dangerous diseases and emerging pathogens.

## 1. Introduction

Laboratories play a crucial role in the rapid detection of infectious pathogens, including endemic, emerging, and re-emerging pathogens and other global health security (GHS) threats. They are essential for syndromic surveillance, early warning system, and the monitoring of response to public health emergencies [1]. In line with these added values, the 8th core capacity of the International Health Regulations (IHR) 2005 requires Member States of the World Health Organization (WHO) to establish mechanisms allowing laboratories to identify and characterize reliably and timely infectious agents and other hazards that may lead to public health emergencies of national or international concern [2,3]. This requirement became more important since the largest Ebola epidemic in West Africa in 2014 and the adoption of the Global Health Security Agenda (GHSA) [4]. This agenda is committed to strengthening, among other components, the capacities of laboratory systems in Africa [5]. Ideally, these laboratory capacities include the ability to confirm pathogens responsible for causing zoonotic diseases. One of the major requirements of global health security is optimal biorisk management in laboratories. In fact, for the work in containment laboratories, including veterinary laboratories, biorisk management (BRM) systems, including adequate biosafety and biosecurity measurements, should be established to prevent the release of or exposure to infectious material [6]. The handling, isolation, storage, and disposal of infectious pathogens pose inherent safety and security risks to laboratories, their staff, the community, the environment, and even the world. As a result, laboratory biosafety and biosecurity systems must be an integral part of any laboratory working with and handling dangerous micro-organisms in order to prevent accidental or intentional release [7]. This might apply to potentially dangerous micro-organisms as well by means of mutation; likewise, seemingly innocuous biological organisms may traverse species and cause disasters. For this purpose, biosafety and biosecurity are increasingly recognized globally as essential concerns for biomedical laboratories, whether the scope of work is clinical, educational, or research-based [8]. To improve the biorisk management, various partners (regional and national) are committed to providing countries with technical and financial supports. Of them, the Food and Agriculture Organization of the United Nations (FAO), the West and Central Africa Veterinary Laboratory Network for Avian Influenza and Other Transboundary Diseases (RESOLAB), and the Regional Network of National Epidemiological Surveillance Systems for Avian Influenza and Other Priority Animal Diseases in West and Central Africa (RESEPI) are committed to strengthening the veterinary laboratory system (VLS) capacities in Africa. They are helping countries to identify gaps in the veterinary laboratory systems and supporting the identification and implementation of corrective measures [5]. FAO has developed two tools, including the laboratory mapping tool safety for the assessment of biorisk management in veterinary laboratories. In Benin, the biological confirmation of zoonotic threats is performed by two veterinary laboratories located in Bohicon and Parakou. The veterinary diagnosis and serosurveillance laboratory (LADISERO) of Parakou is the reference laboratory in the country. This laboratory has the autonomous capacity to confirm some main pathogens, including Rift Valley fever virus (RVFV), avian influenza virus (AIV), rabies virus (RAV), and *Bacillus anthracis*. These pathogens are of great importance for GHS, and effective biorisk management is paramount to avoid or reduce the risk of workers exposure and accidental or intentional use of pathogens. Any weaknesses in the biorisk management in this laboratory can then lead to serious public health events that can compromise national and global health security. However, to our knowledge, there is no external assessment of biosafety and biosecurity management in veterinary laboratories in Benin. Filling this gap is urgent in the current context of emerging and re-emerging epidemics with the identification and implementation of corrective actions. Therefore, as part of the ongoing assessment of the national laboratory system capacities for the detection of GHS infectious threats in Benin, the current study is undertaken with an aim to assess the biorisk management in LADISERO.

## 2. Materials and Method

### 2.1. Settings

**Figure 1:**
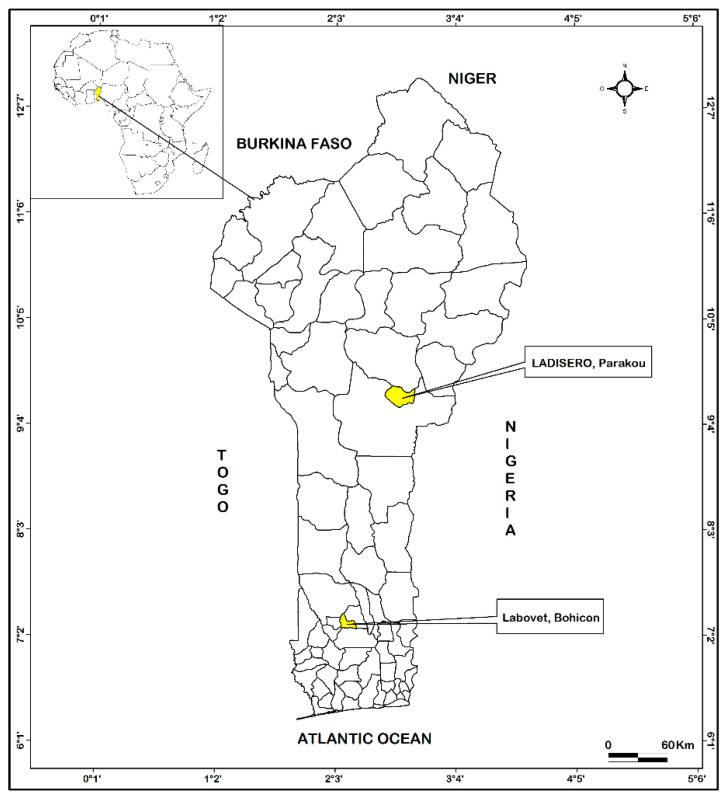
Geographical position of veterinary laboratories in Benin Republic in February 2021.

The study was conducted in the Benin Republic. The country has 12 departments and shares borders with Burkina Faso, Niger, Nigeria, Togo Republics, and the Atlantic Ocean (Figure 1). The country has a veterinary syndromic epidemiological surveillance system for animal diseases supported by laboratory confirmation. In 2017, the Ministry of Health, supported by the livestock Direction and other One Health partners, conducted the risk mapping for priority pathogens, including zoonoses. AIV, *Bacillus anthracis*, and hemorrhagic fever viruses (RVFV, Lassa fever virus, and Ebola virus) were some of the pathogens identified. The country is also threatened by zoonotic pathogens reported in the neighboring countries. Benin’s national VLS is composed of just two public sector laboratories. There is no private or university VL in the country. LADISERO is established in Parakou, in the Borgou department in the northern part of the country; it is the most well-equipped modern laboratory in the country and acts as the national reference laboratory. The equipment available includes equipment for serology (ELISA reader, water bath, refrigerator +2 °C to +8 °C, and freezer −20 °C and −80 °C), bacteriology (incubator, autoclave, and microscope), equipment for conventional polymerase chain reaction tests (thermal cycler, ice machine, and darkroom with disclosure equipment), parasitology (microscope), and rabies unit (immunofluorescence microscopy). Biological safety cabinets (BSC) are available in all sectors except parasitology. The second laboratory is the veterinary laboratory of Bohicon (Labovet) in the Zou department in the central part of the country (Figure 1).

### 2.2. Method

This was a cross-sectional, descriptive, and evaluative study. The study population was the veterinary laboratory system in Benin. The targeted laboratory was the veterinary laboratory of Parakou. The non-probability sampling method was used with a reasoned choice of LADISERO. The choice was guided by the reference role played by the laboratory. Data collection took place from 8 to 9 February 2021. Informed consent from stakeholders was obtained prior to the survey. In the context of the COVID-19 pandemic, strong measures were taken to comply with barrier measures before, during, and after interactions with targeted persons. The FAO Laboratory Mapping Tool-Safety (FAO LMT-S) (http://www.fao.org/ag/againfo/programmes/en/empres/news_130514.html, accessed on 15 January 2019) [9] was used to evaluate the biorisk management of the laboratory. This tool defines 20 categories of criteria covering four areas (Table 1). The main areas are:

Administration;
Operational;
Engineering;
Personal protection equipment (PPE).

Data collection techniques included group discussion completed by observation and document exploitation. The questions in the LMT safety were administrated to the laboratory team during this group discussion. The laboratory manager and staff members present on the days of the survey participated in the discussion. The consensual responses of the team were inserted into the tool. The verification of some key aspects was undertaken to validate the response through observation of the laboratory working environment and exploitation of some documents. The LMT-S has 98 questions ranged from 2 to 8 per category. Each question is rated from 1 to 4 based on the level of achievement in the laboratory. The highest level of compliance and activity receives a score of 4, and the most basic level of activity or awareness receives a score of 1. The tool defines for each question conditions that correspond for 1, 2, 3, or 4 during the rating process [5]. The LMT-S has features that automatically generate the score obtained by the laboratory for each category and domain, as well as the overall average indicator of the laboratory. The tool also calculates a confidence score based on the number of questions that are not applicable or not evaluated by the assessor. Completion of 0–69% of the questionnaire provides a low confidence score, 70–89% a medium confidence score, and 90–100% completion of the questionnaire is ranked as reliable. The confidence score is reported as a component of the summary results [5]. The laboratory safety and biosecurity data are tabulated and also graphically depicted using a color-coded radar-style chart, with rankings allocated to 0–20%, 20–40%, 40–60%, 60–80%, and 80–100% as compared to the optimum 100% benchmark score for each area and category [5]. In our study, the capacity of LADISERO was rated as good if the laboratory had an overall average indicator of at least 80%. If the score was less than 80%, the capacity was judged insufficient. The evaluation of LADISERO was carried out by a team composed of one epidemiologist (V. D. S.) and one veterinarian (A. G. A. A.) who had more than 5 years of experience in VL management. The epidemiologist was affiliated with the Regional Institute of Public Health (IRSP) Ouidah of the University of Abomey-Calavi; the veterinarian was affiliated with the livestock direction. The process was supervised by one professor in bacteriology–virology (D. A.) and two public health professors (P. A. A. and D. E.-M. O.). The results were automatically generated by the features of the FAO LMT-S. 

## 3. Results

The results are presented by area and per category. 

### 3.1. Administration Area

**Figure 2:**
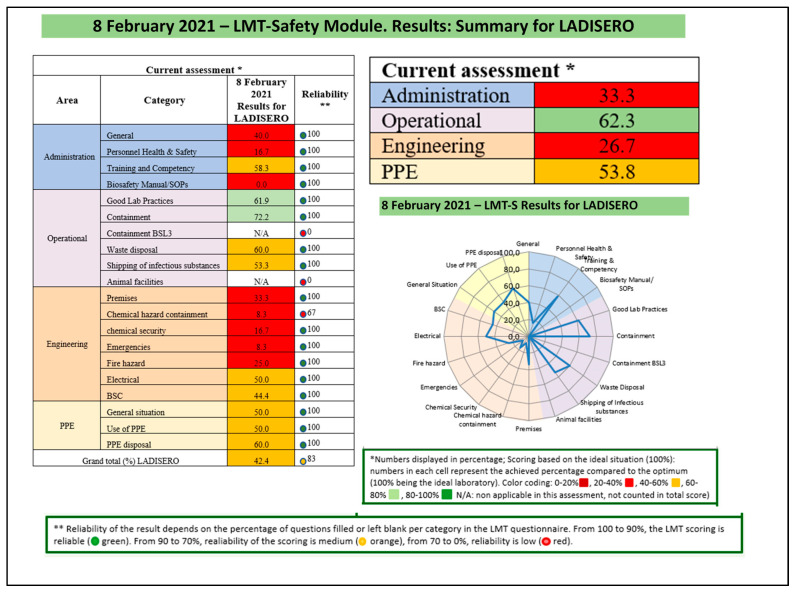
Results of the biosafety and biosecurity assessment in the reference veterinary laboratory of Parakou (LADISERO), Benin, February 2021.

The score per category was ranged from 0% for biosafety manual and standard operating procedures (SOP) to 58.3% for training and competency. The total score for the domain of administration was 33.3% (Figure 2).

### 3.2. General Category

The laboratory has no biosafety and accident prevention program, no security program (policy and procedures) to protect from theft or misuse of selected high-risk pathogenic agents, and no appointed security officer. A biosafety audit was conducted within the last 24 months (by an external auditor or self-audit), but no follow-up has been initiated to correct the problems. There was no risk assessment conducted on the biosafety practices; SOPs are known by the team, but there is no written SOP on biosafety in the laboratory. The laboratory does not maintain a pest control and monitoring program for the control of pest and disease vectors. 

### 3.3. Personnel Health and Security

The laboratory does not monitor staff health. Besides the government insurance mechanism for the permanent government workers, the other staff may seek medical care at their own expense even in case of an accident or laboratory-related diseases. Post-exposure vaccinations or prophylaxis are offered only on request. The laboratory has no emergency documents and no emergency response supplies. There is no formal program or requirement in place for accidents and adverse incidents. Accidents are not systematically reported, and there is no documentation available on accidents that happen in the laboratory.

### 3.4. Training and Competency

Selected staff are trained on biorisk management, and there is a good level of awareness of workers in biosafety. The training addresses the precautions needed for handling specific infectious agents manipulated in the laboratory; these include potential routes of exposure, health risks, signs and symptoms, preventive or control measures, and response to inadvertent exposure. Staff are specifically trained and verified competent before working with specific pathogens and using specific procedures, but there is no training on chemical and biological spill-clean-up. There are no spill kits available; only disinfectants and paper towels are available in the laboratory to clean up chemical and biological spills. 

### 3.5. Biosafety Manual/SOPs

There is no biosafety manual available to the technical staff. The development of the SOP has started, but it is not yet completed.

### 3.6. Operational Area

The operational domain categories score ranged from 53.3% for shipping infectious substances to 72.2% for containment. The score of the operational area in LADISERO was 62.3% (Figure 2). LADISERO is a biosecurity level (BSL) 2 laboratory and does not implement laboratory animal care. Then, animal facility and BSL3 containment capacities were not applicable in the laboratory. 

### 3.7. Good Laboratory Practices

This was the second performing category in the operational area with the score of 61.9%. The laboratory showed clear evidence of good laboratory practices with appropriate signage on rules and regulations and evidence of training for all laboratory and auxiliary staff on these good practices. The personnel apply the guidelines of not storing food, eating, drinking, smoking, applying make-up, or handling contact lenses in work areas. The laboratory is well maintained, but there are no documented SOPs on housekeeping to follow. There is no control of the effectiveness of disinfectants used. Chemical indicators are not used to verify autoclave performances, and all critical biosafety equipment is maintained when the equipment shows signs of wear or failure. 

### 3.8. Containment

Containment was the best performing category (72.2%) in the operational area. The laboratory conducted a risk assessment for biocontainment of all high-consequence pathogens and all biological hazards in line with national regulation in December 2020. Its area has restricted access with signs indicating this restriction. The access to BS-laboratories and freezer rooms is controlled and restricted to approved and authorized staff; doors are lockable. Staff had been trained and verified competent about pathogens for BSL2. However, the training is not regular. The laboratory does not have an annual training plan. The international biohazard pictogram is located on the front of the doors. Each pictogram provides specific information about the targeted risk. All potentially infectious samples with a potential for creating infectious aerosols or splashes are manipulated within a biological safety cabinet (BSC); all staff using a BSC have been trained. The laboratory has no emergency response plan for handling biohazard samples in case of a major facility failure.

### 3.9. Waste Disposal

Disinfection and containment occur in the laboratory, but there are no policies and procedures specific to waste disposal, and the decontamination system has not been validated. An incinerator is available and properly maintained but does not meet the requirements for the laboratory. There is a need for an incinerator with much larger capacity, a more suitable chimney, and a discharge channel compliant with regulations. The laboratory has no chemical waste treatment. The laboratory has an autoclave. Sharps for disposal (e.g., needles, broken glass, etc.) are separated from routine laboratory wastes into hard-shell containers. There are enough and appropriate equipment and disposable materials; infectious and chemical waste disposals are available.

### 3.10. Shipping of Infectious Substances

There is a designated area for specimen reception, but there is no apparent system for the safe distribution of samples or for recording information. One staff member is trained and certified to ship infectious materials according to current national and international regulations. His certificate was updated in 2020. This trained staff member is aware of national and international regulations and has access to current regulations. Instructions for packaging of infectious material are available; shipping containers for local transportation meeting international and national transportation requirements are not available. A list of infectious materials shipped from the laboratory could be generated from courier records. Secondary containers and packaging materials are visually inspected, and if needed, are decontaminated and reused.

### 3.11. Engineering Area

The engineering category scores ranged from 8.3% for chemical hazard containment and emergencies to 50.0% for electrical. Five out of the seven categories had a score of less than 40%.

### 3.12. Premises

The laboratory staff is actively involved in taking samples from the farm or domestic animals without a prior quarantine period. The laboratory does not meet regulatory construction requirements. The laboratory is sorely lacking in adequate infrastructure. The laboratory management is aware but does not apply the regulations for many reasons. The laboratory is old and requires renovations to meet international standards. The laboratory has air conditioners but not enough of them. Noxious odors and fumes are not controlled. The work areas, including benching, are of medium quality. The bench tops are built with tiles. Illumination is sufficiently bright. Each laboratory contains sinks, soaps, and disinfectants for washing hands, but there is no sign or job aid indicating the correct method to wash hands. Each staff member has a locker located outside of the laboratory facility to store their possessions. There is no pathologist in the laboratory.

### 3.13. Chemical Hazard Containment

Chemical substances are inconsistently stored in large volumes with appropriate signage. There is no separation of chemicals. The emergency procedures are not documented, and staff is not trained in actions. There are no personal safety measures for radiation in place. There are no physical safety measures for radiation in place. There is no radiation protection officer, and no appropriate reference manual is available for consultation. Also, there was no radiation spill kit evidenced. 

### 3.14. Chemical Security

Chemical wastes are not treated appropriately from a safety or environmental point of view. There are no SOPs that regulate disposal procedures. Chemicals are properly stored, labeled, and separated. Safety data sheets are accessible but not for every product in every department. There is no chemical safety officer appointed. There is no emergency clean-up kit for chemical spills.

### 3.15. Emergencies

There were no emergency plans at the time of the study. A safety shower is available in some laboratories but with limitations (either not regularly tested or non-functional, or only in cold water etc.). There is no biological spill kit and no emergency eyewash facilities.

### 3.16. Fire Hazard

There is no integrated fire detection system and no automated suppression system installed. Fire alarms are not installed, and there are not regular fire drills. Fire exits are not marked, and there is no fire evacuation plan. Corridors, aisles, and circulation areas are clear and unobstructed for movement of staff and fire-fighting equipment. The laboratory rooms with potential fire hazards are equipped with appropriate extinguishers for an emergency. All staff are properly trained on the use of fire extinguishers. Portable fire extinguishers are not maintained fully charged and in working order, and expiration dates are not checked and respected.

### 3.17. Electrical

Prior to the purchase of electrical equipment, technical specifications are defined to ensure national compliance. However, the laboratory has no control capacity. Electrical equipment is tested but only by visual inspection. There is no SOP in the laboratory to check electrical equipment. A response plan for power failures involving critical biosafety equipment is not available. The interior wiring has a grounded conductor. The sockets are slightly overloaded, although not dangerously. There is no control prior to the purchase of electrical equipment; however, technical specifications are provided to the providers.

### 3.18. Biosecurity Cabinet

The National Sanitation Foundation (NSF) certified accessors did not test the BSC for 5 years or more. The laboratory has no schedule or protocol for thorough cleaning. However, there is a consistent surface cleaning and disinfection practiced; the BSC are used by trained and competent staff and located in adequate rooms. 100% of BSC are in conformity with internationally recognized standards. All tasks that require the usage of BSC are done in the appropriate BSC.

### 3.19. Personal Protective Equipment Area

This area score was 53.9%. Two categories had a score of 50.0%, while the PPE disposal had a score of 60.0%. 

### 3.20. General Situation

The laboratory has a basic minimal PPE requirement for working in the laboratory. Disposable gloves are used with infectious agents and potential toxins, non-disposable gloves are available for dishwashing, autoclaves, and similar hand protection needs. All appropriate PPE is provided without cost for staff use and is individually fit-tested. Staff are trained and competent in PPE don and doff procedures. Technical staff receive initial training on the correct use and removal of PPE.

### 3.21. Use of PPE

Laboratory PPEs are removed and stored or discarded in designated areas whenever leaving the work area of the laboratory. There is no specific requirement for protective eyewear or full-face protection (but they are available) for staff to use for procedures with the potential to generate splashes. Within the work area, gloves are removed before touching common objects, but hands are not always washed. There is no required PPE for working with temperature extremes, including ultralow temperatures.

### 3.22. PPE Disposal

The laboratory has a specific maintenance program for reusable PPE. The cleaning and disinfection of reusable PPE are not done correctly. Protective clothes are decontaminated when applicable prior to being laundered and are laundered by the laboratory on a regular schedule. Sufficient clean replacements are available at all times. Disposable gloves are worn whenever working with potentially toxic or infectious materials and biologicals; they are changed frequently during a work shift and are not reused. Non-reusable PPE are used and disposed of as biohazardous laboratory waste, but they are not treated before disposal. 

### 3.23. Overall Biosafety and Biosecurity Score in LADISERO

The overall average score of LADISERO for the biorisk management was 42.4%, with a medium confidence rate of 83% (Figure 2). The average score is less than 80%. Therefore, the LADISERO biorisk management capacity was insufficient.

## 4. Discussion

The objective of the study was achieved. The biorisk management in LADISERO was assessed, and the different areas of improvement were identified. The combination of different data collection methods enabled a global and objective view of the laboratory biorisk management status and capacity. 

### 4.1. Overall Biorisk Management Capacity

The biorisk management capacity in LADISERO is in need of urgent improvement. This is in direct link with insufficient capacity reported during the assessment. The engineering capacity is the lowest. This was also reported by Mouillé in 17 laboratories of group A as well as in other 17 laboratories of group B of FAO, where the average score of the engineering was the lowest [5]. Chemical hazard containment and chemical security were among the lowest categories as reported in Region A by Mouillé. Also, the emergency preparedness category score in LADISERO was the lowest in the engineering area, as reported in region B of FAO [5]. The administration was the second-lowest area. This was also reported in the two FAO regions, where the average score for the administration was the lowest after the engineering area [5]. LADISERO does not have any biosafety manual or SOP. Although weaknesses were reported in this category in other laboratories, the level of performance is worrisome. The workers declare that they know the procedures, but there is no document to ensure that principles are being followed up. Aware of this weakness, the LADISERO team has initiated the development of standard manuals and SOP on biorisk management. There is an urgent need to finalize and validate this important document. Personal health and safety is the second weak category in the administration area. Mechanisms for the protection of laboratory workers are not established or fully functional. The workers are then operating in an insecure work environment that can hinder their motivation. Some of them can benefit from government insurance, but this does not fit with the emergency mode needed in such circumstances. While PPE was the best performing area reported in the two FAO regions in the Mouillé study, it was the second performing area in LADISERO, but the score of PPE in LADISERO is higher than the average score reported in region A and region B [5]. On the other hand, the PPE disposal practices seem to be better in LADISERO (60%) compared to the average score in FAO region B (50%) [5]. The operational is the best performing area in LADISERO. The laboratory score is higher than the average score reported in Region A and in Region B of FAO [5]. The laboratory scores for each category are higher than 50 %. Laboratory good practices and containment are the best performing categories. The laboratory shipping of infectious disease score is higher than the average score in FAO region B [5]. The laboratory performance in operation can be explained by its equipment and its status as a reference laboratory in Benin. On the other hand, the limited number of veterinary laboratories in Benin requires the laboratory outreach missions for samples collection and transport; this obligates the laboratory to improve capacities for biorisk prevention during samples collection, packaging, and transport. Despite the absence of standard manuals and SOPs, the lab workers are doing their best to apply the standard in biorisk management.

The overall average score of the laboratory for biorisk management is weak. However, the score of 42.9% is higher than the 41.3% reported in the FAO region A and the 28.1% reported in region B [5]. The LADISERO’s relatively better performance can be explained by its status as a reference laboratory, its equipment, and the support from partners. However, LADISERO’s overall performance score is lower than the maximum score reported in FAO region A (64%) and region B (77%). This means that the laboratory can do better and must do better, especially as it acts as a reference laboratory. The findings in LADISERO about biorisk management seems to be largely reported in other veterinary laboratories in Africa and other regions. This is probably due to insufficient perception of the importance of the veterinary laboratories, the low commitment to the improvement of veterinary laboratory capacities, and the insufficient budget allocated to these laboratories. The role of veterinary laboratories in the GHS is not yet commonly perceived and promoted. Focus is still largely put on the health system without proper improvement of the One Health approach. A high proportion of laboratories lack adequate biorisk management materials and principles, putting workers and the entire communities in uncomfortable work conditions. The training on biosecurity is at different levels of achievements in West Africa, with some countries more advanced [10]. Advocacy toward national governments and partners, especially One Health approach stakeholders, is urgent to improve the safety capacity of the veterinary laboratories. 

### 4.2. Biosafety and Biosecurity

The biosafety capacity in LADISERO is weak. The working conditions described above can explain this status. The laboratory is lacking procedures, training, supervision, and key logistics for biosafety. This situation is opposite to the situation reported in 18 veterinary laboratories in Europe. The results suggest that the biorisk management elements referring to standard microbiological working practices and the handling of infectious material were fulfilled particularly well [6]. In LADISERO working conditions, the workers of the laboratory are at high risk of contracting a laboratory-acquired infection (LAI) [11]. The absence of SOP and supervision can easily lead to human error identified as the main cause of LAI [12]. Curiously, there are no documentation mechanisms on LAI. This absence of documentation does not mean that there is no LAI in the laboratory. In fact, while there were no data in Pakistan on LAI, a survey conducted revealed six cases of individuals with known LIAs [13]. Contrary to the observations made in Pakistan with a lack of recognition for employees’ rights and benefits in the workplace for biosafety, the LADISERO managers and workers are aware of the importance of biosafety as their rights [8]. Despite the insufficient working conditions, they are handling dangerous pathogens such as Bacillus anthracis, AIV, and RVFV. These pathogens are the main threats to GHS. Therefore, the risk is not only limited to the laboratory workers but further extends to the national and international community. If laboratory workers contract an infection, they can easily contaminate their relatives and neighbors. The absence of emergency supplies in the laboratory and of the formal program for accidents and adverse incidents can worsen this situation. The biosafety level in LADISERO is similar in many other veterinary laboratories across African region [5]. This constitutes a major threat to GHS. Adequate measures should be implemented by partners, governments, and laboratories’ managers to improve the biosafety capacities. Another urgent aspect in LADISERO is the improvement of the biocontainment capacities. Although the access to the laboratory and key areas is restricted, it is urgent to ensure adequate measures to improve the biosecurity protocols and practices in the laboratory. 

## 5. Conclusions

Despite the progress made, there is still a need to improve the biorisk management system in LADISERO. The required procedures are not yet in place, as well as personnel training and supervision mechanism. There is a lack of human, financial, and logistical resources, worker health and security mechanisms. However, some technical areas, including the operational and the PPE areas, appear to have better performance indicators despite missing resources. The laboratory presents some assets that can contribute to the improvement of biosafety and biosecurity. The appointment of a biorisk management officer, the quick finalization of the standard procedures, the increase of the laboratory budget, and the development and implementation of the biorisk management improvement plan appear as main actions to improve the biorisk management as part of the GHS in Benin.

## Figures and Tables

**Table 1:** Area, categories, and number of questions per category for the FAO laboratory mapping tool.

Area	Category	Number of Questions
Administration	General	5
	Personnel health and safety	4
	Training and competency	4
	Biosafety manual/Standard operating procedures (SOPs)	2
Operation	Good lab practices	7
	Containment	6
	Containment BSL3	8
	Waste disposal	5
	Shipping of infectious substances	5
	Animal facilities	7
Engineering	Premises	7
	Chemical hazard containment	6
	Chemical security	4
	Emergencies	4
	Fire hazard	4
	Electrical	4
	Biological safety cabinet (BSC)	3
Personal protective equipment (PPE)	General situation	4
	Use of PPE	4
	PPE disposal	5

## Data Availability

The data that support the findings of this study are available from the corresponding author, V. D. S., upon reasonable request.
